# The influence of anti-CD20-directed B cell depletion across different agents on Epstein-Barr virus-specific humoral and cellular immunity in patients with multiple sclerosis

**DOI:** 10.1186/s12974-026-04062-0

**Published:** 2026-09-28

**Authors:** Jakob Stögbauer, Saskia Bronder, Niklas Saenz Kämpfer, Celina Kron, Gabriel Gonzalez-Escamilla, Katharina Schwartz, Beatrice Tocariu-Krick, Eszter Nemeth, Wenlin Hao, Chiara Huber, Sven G. Meuth, Olaf Stüve, Rebecca Urschel, Tina Schmidt, Sergiu Groppa, Martina Sester, Mathias Fousse

**Affiliations:** 1 Department of Neurology, Saarland University Medical Center, Homburg, 66421 Germany; 2 Department of Neurology, Saarland University, Building 90, Kirrberger Straße, Homburg, 66421 Germany; 3 Department of Transplant and Infection Immunology, Saarland University, Homburg, 66421 Germany; 4 Department of Neurology, University Hospital Münster, Münster, Germany; 5 Department of Neurology, University of Texas Southwestern Medical Center, 5323 Harry Hines Blvd, Dallas, TX 75390-9096 USA; 6 Center for Gender-Specific Biology and Medicine (CGBM), Saarland University, Homburg, Germany

**Keywords:** Multiple sclerosis, Epstein Barr virus, T cells, B cell depletion

## Abstract

**Background:**

Epidemiological evidence has established a central role of Epstein-Barr virus (EBV) in the pathogenesis of multiple sclerosis (MS). While EBV-specific T-cell responses may influence disease progression, the impact of anti-CD20 B-cell depletion therapy (BCDT) on EBV-specific cellular and humoral immunity remains insufficiently characterized, particularly in comparison with healthy controls (HC), untreated people with MS (pwMS), and pwMS on different BCDT agents.

**Main body:**

Methods

In this cross-sectional study, pwMS receiving BCDT, untreated pwMS at initial diagnosis (MS-ID), and age- and sex-matched HC were enrolled. EBV-specific and polyclonal CD8^+^ and CD4⁺ T-cell responses were quantified and functionally characterized following stimulation with a EBV-derived peptide-mix predominately containing MHC class I binding peptides or Staphylococcus aureus enterotoxin B (SEB) using multiparametric flow cytometry. Anti-EBNA1- and anti-VCA-IgG levels were measured by ELISA.

**Results:**

A total of 183 participants were included: 54 pwMS receiving BCDT (mean age 47.0±15.6 years; 31 females), 107 controls (47.2±18.3 years, 54 females), and 22 MS-ID (33.4±11.1 years, 20 females). EBV-specific CD8⁺ and CD4⁺ T-cell levels were similar in controls and MS-ID. In contrast, BCD-treated pwMS showed significantly lower frequencies of EBV-specific CD8⁺ (*p*<0.0001) and CD4⁺ T cells (*p*=0.0004) than controls, and lower EBV-specific CD8⁺ T-cell levels than MS-ID (*p*=0.0023). In contrast, polyclonally stimulated CD8⁺ T-cell responses were largely comparable across groups, indicating preserved polyclonal T-cell function. EBV-specific CD8⁺ T cells in both patients and controls were predominantly polyfunctional, coexpressing IFNγ, IL-2, and TNF. Longer BCDT duration was associated with lower frequencies of EBV-specific CD8⁺ T cells (*p*=0.0003). Ofatumumab-treated patients exhibited higher EBV-specific CD8⁺ T-cell frequencies than ocrelizumab-treated patients (*p*<0.0001). Despite BCDT, anti-EBNA1- and anti-VCA-IgG levels were significantly higher in pwMS than in MS-ID or controls, and were unrelated to treatment duration, disease duration, disability, or BCDT agents.

**Conclusions:**

Unlike in treatment-naïve pwMS, BCDT is associated with selectively lower EBV-specific T-cell frequencies while EBV-specific and polyclonal T-cell functionality was largely preserved. In contrast, EBV-specific antibody levels were persistently higher despite treatment. Given the remarkably high clinical efficacy of BCDT in this cohort, the findings support a prominent role of EBV-specific cellular immunity in ongoing MS pathophysiology and suggest that reduced EBV-reactive T-cell responses may contribute to this potency. Differences between BCDT agents warrant further investigation.

**Supplementary Information:**

The online version contains supplementary material available at https://doi.org/10.1186/s12974-026-04062-0.

## Background

Multiple sclerosis (MS) is a chronic inflammatory disease of the central nervous system (CNS) and the most common non-traumatic cause of disability in young adults [1]. Despite significant advancements in diagnosis and treatment of the disease, the etiology of the condition remains uncertain. It is widely presumed to be the result of a multifactorial pathogenesis, involving genetic susceptibility, lifestyle factors such as obesity in adolescence and tobacco use, as well as environmental factors such as vitamin D intake and viral infections [2, 3]. Within the latter context, infection with the Epstein-Barr virus (EBV) plays the central role [4]. Since at least 1980, epidemiological studies have indicated an association between EBV infection and the development of MS [5–7]. Recent data from large cohorts have renewed the debate describing a 32-fold increased risk of developing MS in the event of EBV seroconversion [8]. At present, given the possibility of misdiagnosis and technical uncertainties, the existence of ‘EBV-negative MS’ is considered virtually impossible; the infection appears to be a prerequisite for the disease [9, 10].

When considering EBV-specific immunity, it is essential to distinguish between specific humoral and cellular immune responses. Following infection, antibodies may develop against various viral components, in particular against the viral capsid antigen (VCA) and EBV nuclear antigen 1 (EBNA1) [11]. Substantial evidence supports a link between humoral immunity towards EBV and MS, as EBNA1-IgG levels are significantly elevated in people with MS (pwMS) compared with healthy controls [12], and higher anti-EBNA1 titers have been demonstrated to increase the risk of developing MS [13]. In contrast, the role of EBV-specific T-cell immunity is less well defined. Increased levels of EBV- or EBNA1-specific CD8^+^ T cells were reported in pwMS and in patients diagnosed with clinically isolated syndrome [14, 15], and more recent data demonstrated approximately twofold higher levels of CD4^+^ T cells to lytic EBV antigens in pwMS as compared to controls [16]. Additionally, the CD8^+^ T cell response may be ineffective in pwMS, who present with a broader MHC-I-restricted EBV-specific T-cell receptor repertoire [17]. Together these findings support the concept of altered EBV-specific cellular immunity in MS. However, further studies integrating quantitative and functional characterization of EBV-specific T cells and the impact of contemporary immunomodulatory therapies are needed to elucidate their role in MS pathophysiology and disease progression [18].

The role of immunomodulatory therapies on EBV-specific immunity in pwMS remains incompletely understood. Particular attention has focused on anti-CD20-directed B-cell depleting therapy (BCDT), as CD20^+^ B cells are considered the primary reservoir of EBV in humans. Although the available evidence is limited and largely based on small cohorts, current studies suggest a reduction in EBV-specific antibodies, in total immunoglobulin G (IgG) [19–21] as well as decreased EBV-specific CD8^+^ T cells [22, 23]. These effects have been proposed as a potential mechanism underlying the remarkable clinical efficacy of BCDT in MS [24]. However, substantial knowledge gaps remain. Most existing studies are limited in sample size and rarely include matched control cohorts or treatment-naïve individuals with newly diagnosed MS. Furthermore, the association between humoral and cellular EBV-specific immune responses has not been systematically analyzed. Moreover, current evidence is almost exclusively restricted to ocrelizumab (OCR), whereas corresponding data on the newer agents ofatumumab (OFA) and ublituximab (UTX), as well as comparisons between these therapies are lacking. Additionally, the functional consequences of BCDT on T-cell immunity remain poorly characterized. To date, only limited evidence points to impaired T-cell reactivity following polyclonal stimulation [25], and analyses of EBV-specific T-cell function have largely been confined to interferon gamma (IFNγ) release, whereas studies on polyfunctional cytokine profiling including interleukin-2 (IL-2) and tumor necrosis factor (TNF) are still lacking.

The objective of the present study was to provide a comprehensive assessment of EBV-specific humoral and cellular immunity in pwMS undergoing BCDT, in comparison with healthy control subjects and untreated pwMS at initial diagnosis. The study placed particular emphasis on the effect of different B-cell depleting agents on cellular and humoral immunity, and on functional profiling of EBV-specific T cells.

## Main text

### Materials and methods

#### Study population

In this observational study, patients suffering from relapsing (RMS) and primary progressive multiple sclerosis (PPMS) were enrolled cross-sectionally between October 2025 and January 2026 at the Department of Neurology of Saarland University Hospital (Homburg/Saar, Germany). At the time of enrolment, all patients were undergoing treatment with an anti-CD20-directed monoclonal antibody (OCR, OFA or UTX). The respective dosage of the preparations was selected in accordance with the summary of product characteristics (OCR: 600 mg intravenously or 920 mg subcutaneously semi-annually; UTX: 450 mg intravenously every 24 weeks; OFA: 20 mg subcutaneously every 4 weeks). Demographic and disease-specific data were extracted from the medical records. Age- and sex-matched controls were recruited among hospital staff and patients with neurological disorders without any underlying immunological condition. Moreover, a cohort of untreated people with MS at the time of their initial diagnosis was enrolled between January and June 2026 as an additional control group. One heparinized blood sample (4.7 ml) per person was collected to analyse EBV-specific humoral and cellular immunity. Only individuals without clinical evidence of EBV infection/reactivation or other herpesvirus infections at the time of analysis were included.

The study was approved by the ethics committee of the Saarland Medical Association (identification number 80/25 and 99/24). Written informed consent was obtained from all patients and control subjects who participated in the study.

#### Determination of virus-specific antibodies

EBV-specific IgG antibodies towards EBNA1 and VCA were quantified using enzyme-linked immunosorbent assay kits according to the manufacturer’s instructions (ELISA; anti-EBNA1-IgG and anti-VCA-IgG). Samples above 200 RU/ml were further tested at a 1:5 dilution (Euroimmun, Lübeck, Germany). The lower limit of detection was 2 relative units (RU/ml). Samples < 16 RU/ml were scored negative, ≥ 16 and < 22 RU/ml were scored intermediate, and ≥ 22 RU/ml were scored positive. Likewise, respiratory syncytial virus (RSV)- and cytomegalovirus (CMV)-specific IgG antibodies were determined using ELISA kits from Euroimmun using the same assay principle and lower limits of detection as for EBV-specific ELISA kits.

#### Quantification of EBV-specific CD4^+^ and CD8^+^ T cells

EBV-specific T cells were quantified directly from whole blood after a 6 h stimulation as previously described for other antigen specificities [26–28]. In brief, heparinized blood was stimulated with 2 µg/ml peptides derived from selected proteins of EBV (PM-pan-EBVselect, jpt Berlin, Germany) predominately containing MHC class I binding peptides. The composition and EBV-antigen distribution of peptides is shown in Supplementary Table (1). The human MHC class I and MHC class II alleles targeted by the peptides are listed in Supplementary Table (2). Stimulation was carried out in the presence of co-stimulatory antibodies against CD28 and CD49d (clone L293 and clone 9F10, 1 µg/ml each). In addition, stimulation with 0.64% DMSO and 2.5 µg/ml of *Staphylococcus aureus* enterotoxin B (SEB; Sigma) served as a negative and positive control, respectively. Moreover, SEB-reactive T cells were analyzed to distinguish characteristics of EBV-specific T cells from polyclonal effector/memory T cells with specificities largely different from EBV. After stimulation, cells were immunostained using fluorescently labelled antibodies towards CD45, CD3, CD4, CD8, CD69, IFNγ, IL-2, TNF (antibodies used are listed in Supplementary Table 3) and analyzed using flow cytometry (BD FACSCanto II, BD FACSymphony A3) and FACSDiva software (BD). Results from samples analyzed on the two different flow cytometers did not differ (Supplementary Fig. 1). EBV-reactive T cells were identified as activated CD69-positive T cells producing IFNγ. Levels of reactive CD4^+^ or CD8^+^ T cells after control stimulation were subtracted from those obtained after EBV-specific stimulation, and 0.03% (CD4^+^) or 0.06% (CD8^+^) of reactive T cells was set as detection limit as defined before based on the variability of negative control stimulation [26]. Moreover, the lower limit of detection was evaluated against reactive CD4^+^ and CD8^+^ T cells in EBNA-1 and VCA-IgG seronegative individuals. Functionality of specifically activated cells were further characterized by cytokine-profiling analyzing individual or combined expression of IFNγ, TNF and IL-2, after Boolean gating, which allowed distinction of seven subpopulations. The gating strategy is shown in Supplementary Fig. 2.

#### Statistical analysis and software

Descriptive statistics are presented using median and interquartile ranges or ranges between minimum and maximum. Comparisons of non-parametric data between two or more than two groups were performed using the Mann-Whitney *U* test or the Kruskal-Wallis test with Dunn’s multiple comparisons test, and the Wilcoxon matched pairs test for paired analyses. Categorical data between two or more than two groups were analyzed with Fisher’s exact test and Chi Square test, respectively. Correlations were calculated according to Spearman. Simple and multiple linear regression was calculated using R² as regression coefficient. Statistical analysis was deemed significant if the p-value was less than 0.05, and confidence intervals were set at 95%.

GraphPad Prism 11.0.2 (GraphPad, San Diego, CA, USA) and SPSS statistics 29.0.2.0 (IBM, Armonk, NY, USA) were used for statistical analyses and preparation of figures. Microsoft Word was utilized for word processing, and Microsoft Excel was employed for the management of raw data tables.

## Results

### Study population of healthy controls and pwMS on B-cell depleting therapy

In the first step, a total of 161 study participants were enrolled including 54 pwMS on B-cell depleting therapy (MS-BCD, 31 females, 23 males) and 107 controls (54 females, 53 males; Table 1). Both groups were matched regarding age and sex. Among patients, 89% were diagnosed with RMS and 11% with PPMS, respectively. At the time of enrollment, the median duration of illness was 9.1 (IQR 15.2) years. BCDT had been established for a median of 1.7 (IQR 3.7) years, and B-cell depleting agents included OCR (n = 26), OFA (n = 20), and UTX (n = 8). The efficacy of BCDT in RMS patients was remarkably high. Since the commencement of treatment, there was no worsening of the EDSS score (median ΔEDSS = 0.0 (range − 1-2.5)) nor any emergence of new T2 lesions on MRI (median = 0 (range 0–4)), and a median annual relapse rate of 0 (range 0–2, Table 1). All but 3 pwMS had complete depletion of peripheral CD19+ B cells, and median serum IgG was within normal ranges (919 (IQR 377) mg/dl, Table 1).

**Table 1:** Clinical characteristics of the study population

	Controls	MS-BCD	*p*-value
	*n* = 107	*n* = 54	
Years of age, mean (± SD)	47.2 ± 18.3	47.0 ± 15.6	0.960^3^
Sex, *n* (%)^a^			
female	54 (50.5)	31 (57.4)	0.504^4^
male	53 (49.5)	23 (42.6)	
Diagnosis, *n* (%)			
RMS	n.a.	48 (88.9)	
PPMS	n.a.	6 (11.1)	
Medication, *n* (%)^b^			
Ocrelizumab (OCR)	n.a.	26 (48.1)	
Ofatumumab (OFA)	n.a.	20 (37.0)	
Ublituximab (UTX)	n.a.	8 (14.8)	
EDSS, median (IQR)			
all patients	n.a.	3 (4.0)	
RMS	n.a.	3 (3.8)	
PPMS	n.a.	6 (3.3)	
Disease duration in years, median (IQR)^c^	n.a.	9.1 (15.2)	
Duration of BCDT in days, median (IQR)^c^	n.a.	623 (1357)	
Outcome measures, median (range^1^			
ΔEDSS since start of BCDT	n.a.	0.0 (-1–2.5)	
Annual relapse rate	n.a.	0 (0–2)	
New T2 lesions since start of BCDT	n.a.	0 (0–4)	
Peripheral CD19^+^ B cells^2^,			
Median % (range) of lymphocytes	n.d.	0 (0–4)	
Serum IgG in mg/dl, median, IQR	n.d.	919 (377)	

^1^RMS patients only; ^2^all patients had complete depletion of peripheral CD19^+^ B cells except three, who had 1, 2 and 4% B cells, respectively; BCDT, B-cell depleting therapy; EDSS, expanded disability status scale, MS-BCD, people with multiple sclerosis on B-cell depleting therapy; PPMS, primary progressive multiple sclerosis; RMS, relapsing multiple sclerosis; n.a. not applicable, n.d. not determined; ^3^unpaired t-test; ^4^Fisher´s exact test

### Higher levels of anti-EBNA1-IgG and anti-VCA-IgG levels in pwMS receiving BCDT

**Fig. 1:**
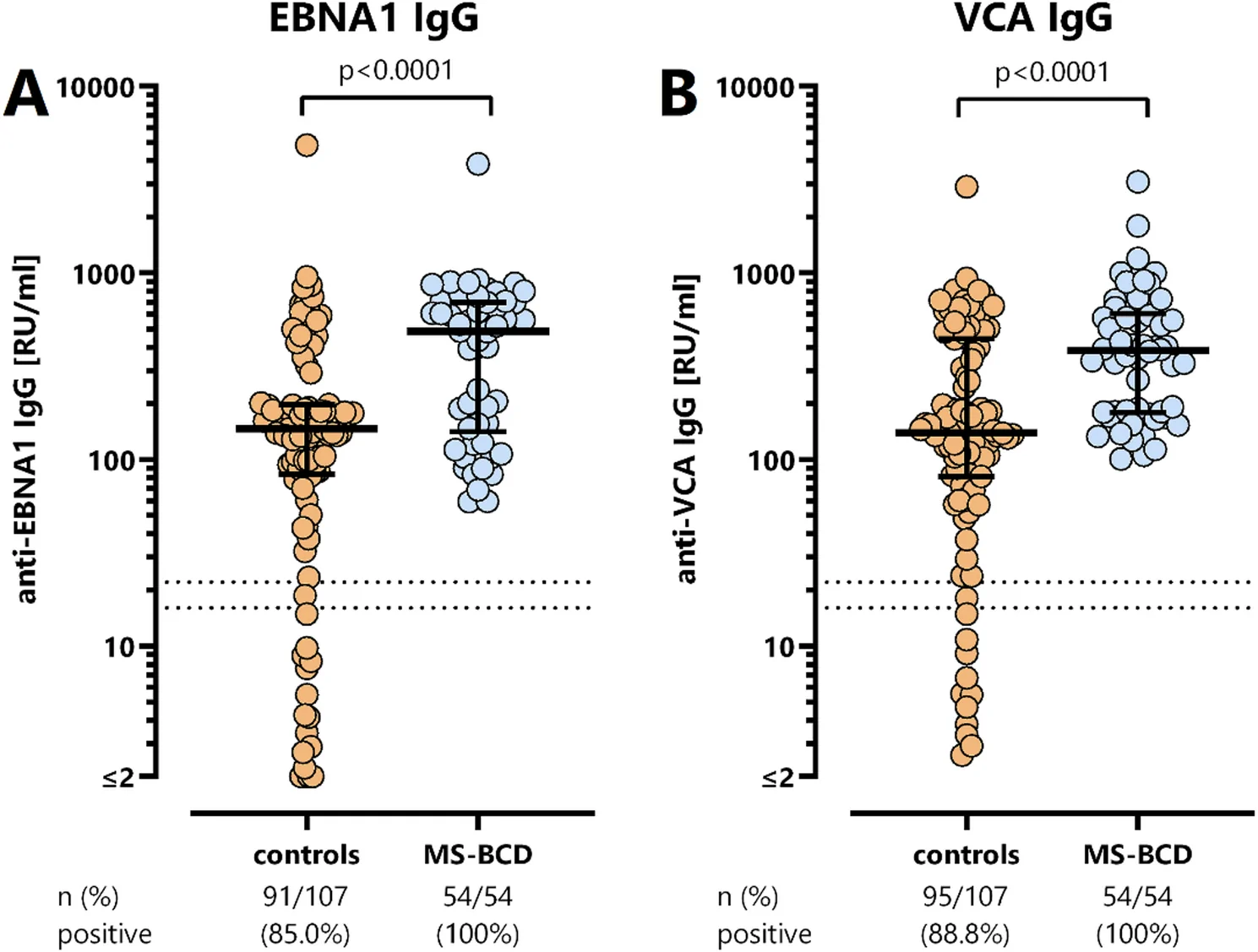
Higher levels of anti-EBNA1-IgG and anti-VCA-IgG in pwMS on BCDT. Levels of (**A**) anti-EBNA1- and (**B**) anti-VCA-IgG antibodies were compared between controls and pwMS on BCDT (MS-BCD). Each point represents one participant. The black lines refer to median and interquartile ranges. Antibody levels are calculated in relative units per ml. Numbers on the bottom refer to the number and percentage of individuals with antibody levels above the detection limit for positivity as indicated by the dotted lines referring to positive, intermediate and negative titers. Groups were compared using Mann-Whitney *U* test. BCDT, B-cell depleting therapy; EBNA1, EBV Nuclear antigen 1; IgG, Immunoglobulin G; pwMS, people with multiple sclerosis; VCA, virus capsid antigen

All individuals with MS under BCDT showed seropositivity for EBNA1 and VCA, while controls were seropositive in only 85% and 88.8% of cases, respectively (Fig. 1). Nine controls (8.4%) were seronegative for both EBNA1 and VCA-IgG. Median levels of anti-EBNA1-IgG and anti-VCA-IgG antibodies were significantly higher in pwMS with BCDT than in controls (EBNA1: 485 (IQR 554) RU/ml vs. 146 (IQR 113) RU/ml; VCA: 383 (IQR 428) RU/ml vs. 139 (IQR 361) RU/ml, each *p* < 0.0001). These differences remained statistically significant when the analyses were restricted to RMS patients only (each *p* < 0.0001), and when data were compared to seropositive controls only (*p* = 0.0008 for EBNA-1-IgG (*n* = 91/107); *p* < 0.0001 for VCA-IgG (*n* = 95/107), Supplementary Fig. 3A and B). We also tested antibody titers towards RSV and CMV as common environmental and persistent viruses, respectively. Most individuals were RSV-IgG positive and less than 50% of individuals were CMV-IgG positive. Unlike EBV-specific antibody levels, RSV- and CMV-specific IgG did not differ between controls and pwMS on BCDT (*p* = 0.359 for RSV-IgG; *p* = 0.613 for CMV-IgG, Supplementary Fig. 3C and D), which emphasizes that the observed difference in EBNA1- and VCA-IgG antibody titers is specific to EBV-associated immune responses rather than reflecting a generalized enhancement of antiviral humoral immunity in pwMS on BCDT.

Among pwMS on BCDT, there was a trend toward a correlation between concentrations of total serum IgG and levels of anti-EBNA1-IgG (*p* = 0.055), but not with anti-VCA-IgG (*p* = 0.435, Table 2). Moreover, specific antibody levels did not show any significant correlations to duration of BCDT (EBNA1: *p* = 0.882, VCA: *p* = 0.171) or disease duration (EBNA1: *p* = 0.862, VCA: *p* = 0.991), nor to EDSS (EBNA1: *p* = 0.734, VCA: *p* = 0.910, Table 2).

**Table 2:** Correlation of clinical variables with EBV-specific cellular and humoral immune parameters in pwMS on BCDT

Variable	EBV-specificCD8^+^ T cells		EBV-specificCD4^+^ T cells			anti-VCA-IgG		anti-EBNA1-IgG	
	R^2^	*p*-value	R^2^		*p*-value	R^2^	*p*-value	R^2^	*p*-value
Duration of BCDT^1^	0.230	**0.0003**	0.015		0.371	0.036	0.171	0.0004	0.882
Duration of disease^1^	0.031	0.210	0.002		0.765	0.000002	0.991	0.0006	0.862
	r	p-value	r		p-value	r	p-value	r	p-value
EDSS^2^	-0.296	**0.033**	-0.077		0.579	0.016	0.910	0.047	0.734
Total IgG^2^	0.280	**0.045**	-0.006		0.965	0.108	0.435	0.263	0.055

^1^R^2^ and *p*-values were calculated by simple linear regression analyses; ^2^r and *p*-values were calculated by correlation analyses according to Spearman. Duration of BCDT was used in days; duration of MS disease was used in years, calculations were performed with log(10) transformed values of EBV-specific CD4^+^ and CD8^+^ T cells, and EBNA1 and VCA IgG; BCDT, B-cell depleting therapy; EDSS, expanded disability status scale

### Lower levels of EBV-specific T-cells in pwMS receiving BCDT

EBV-specific CD8^+^ and CD4^+^ T cells were quantified as CD69^+^ IFNγ^+^ T cells following stimulation with the selected peptide mix (Supplementary Table 1) after subtraction of the negative control stimulus DMSO. The nine EBNA-1/VCA-IgG seronegative controls had similarly low levels of reactive cells in both stimuli, and EBV-specific CD8^+^ and CD4^+^ T cells well below the pre-established lower limit of detection of 0.06% and 0.03% (supplementary Fig. 4A). Raw percentages of reactive CD8^+^ and CD4^+^ T cells in EBNA1/VCA-IgG seropositive individuals are shown in supplementary Fig. 4B and C, respectively.

**Fig. 2:**
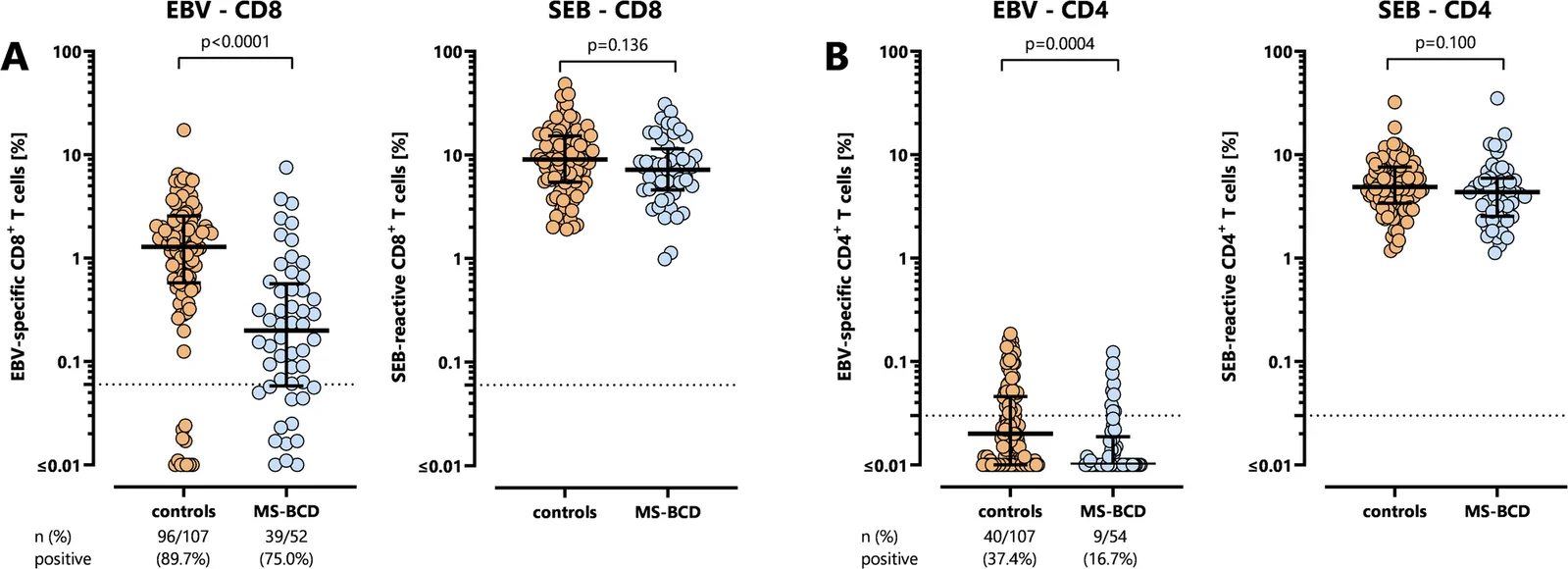
Lower levels of EBV-specific CD8 + and CD4 + T cells in pwMS on BCDT than in controls. EBV-specific and SEB-reactive (**A**) CD8^+^ T cells and (**B**) CD4^+^ T cells were compared between controls and pwMS on BCDT (MS-BCD). Each point represents T-cell frequencies of one participant. The black lines refer to median and interquartile ranges. Numbers on the bottom refer to the number and percentage of individuals with reactive CD4^+^ or CD8^+^ T cells above the respective lower limits of detection, which are indicated by the horizontal dotted line (0.03% for EBV-specific CD4^+^ T cells and 0.06% for EBV-specific CD8^+^ T cells, respectively; EBV-specific CD4^+^ and CD8^+^ T-cell levels from EBNA1/VCA IgG seronegative individuals (*n* = 9) were well below these detection limits, see Supplementary Fig. 4A). Levels of CD8^+^ and CD4^+^ T cells toward EBVpan peptide mix and the polyclonal stimulus SEB were compared between the groups using Mann-Whitney *U* tests. The samples of two pwMS were excluded from the EBV-specific CD8 T-cell analyses as indeterminate due to high non-specific background reactivity in the control stimulation. Samples from controls were in part analyzed on a FACS Canto II, whereas all other samples were analyzed on a FACSymphony A3 flow cytometer. Results from samples analyzed on the two different flow cytometers did not differ (Supplementary Fig. 1). BCDT, B-cell depleting therapy; IFNγ, interferon gamma; pwMS, people with multiple sclerosis; SEB, *Staphylococcus aureus* enterotoxin B

When considering both controls and pwMS on BCDT, frequencies of EBV-specific CD8^+^ T cells were detectable in 135 out of 159 samples (84.9%, with 2 samples scored as indeterminate). When the groups were compared, pwMS the MS-BCD group had significantly lower median specific CD8^+^ T-cell levels than controls (0.20% (IQR 0.51%) vs. 1.29% (IQR 1.97%), *p* < 0.0001, Fig. 2A). When comparing MS-BCD patients with RMS and PPMS, numerically higher median CD8^+^ T-cell frequencies were observed in the RMS group (0.23% (IQR 0.53%), *n* = 48) than in the PPMS group (0.03% (IQR 0.42%), *n* = 5), but this difference did not reach statistical significance (*p* = 0.117). Nevertheless, the difference between patients and controls remained significant, when the analysis was restricted to patients with RMS only (*p* < 0.0001) or when data were compared to EBNA-1/VCA-IgG seropositive controls only (*n* = 98, *p* < 0.0001, Supplementary Fig. 5A). The observed differences were specific to EBV-reactive T cells, as no distinction in reactivity of CD8^+^ T cells was detected following polyclonal stimulation with SEB (*p* = 0.136, Fig. 2A).

A smaller fraction of peptides in the peptide pool (12/135) used for stimulation was targeted to be presented in MHC class II molecules thereby stimulating CD4^+^ T cells. As expected from this composition, EBV-specific CD4^+^ T-cell levels detected after stimulation with this peptide pool were generally low in both groups (Supplementary Fig. 4 and Fig. 2B). A lower proportion of pwMS on BCDT had EBV-specific CD4^+^ T-cell frequencies above the lower limit of detection (40/107 versus 9/54, *p* = 0.007), and frequencies were lower in patients (0.01% (IQR 0.02%)) as compared to controls (0.02% (IQR 0.04%), *p* = 0.0004), but these results should be considered as exploratory, as most individuals in both groups had CD4^+^ T-cell levels below the lower limit of detection. SEB-reactive CD4^+^ T-cell levels did not differ between the groups (*p* = 0.100; Fig. 2B).

EDSS-scores were inversely correlated with EBV-specific CD8^+^ T-cell levels (Spearman *r*=-0.296, *p* = 0.033), and a weak correlation was found between total IgG and EBV-specific CD8^+^ T-cell levels (*r* = 0.280, *p* = 0.045, Table 2). Likewise, a longer duration of treatment showed a significant association with lower EBV-specific CD8^+^ T-cell frequencies (R^2^ = 0.230, *p* = 0.0003), whereas duration of disease did not (R^2^ = 0.031, *p* = 0.210). No significant associations were observed with CD4^+^ T-cell responses (Table 2).

### Differential effect of BCDT agents on EBV-specific humoral and cellular immunity

**Fig. 3:**
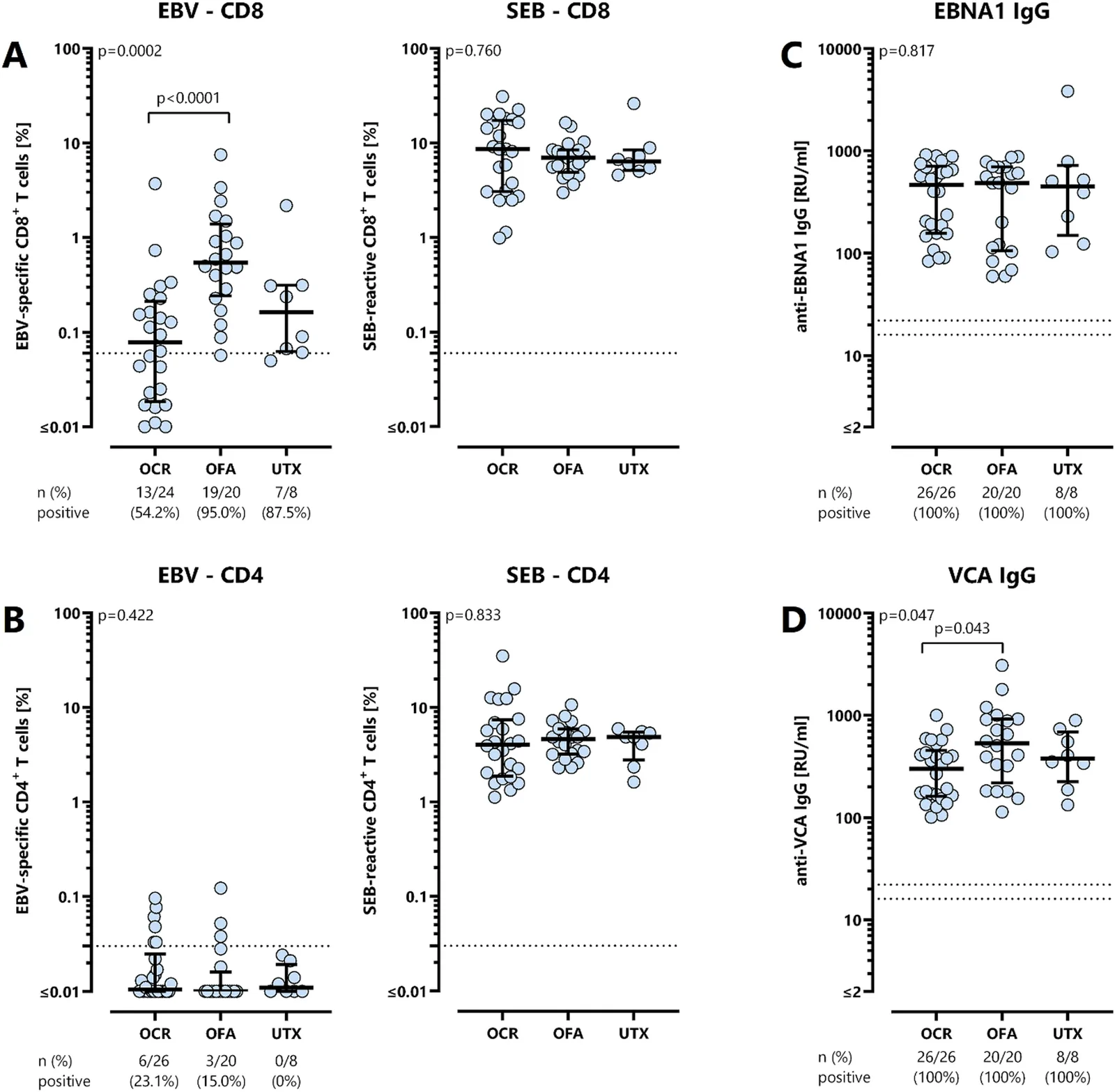
Lowest levels of EBV-specific CD8^+^ T cells in pwMS on OCR. The levels of EBV-specific and SEB-reactive (**A**) CD8^+^ and (**B**) CD4^+^ T cells, as well as (**C**) EBNA1- and (**D**) VCA-specific IgG-levels are stratified for people with MS undergoing treatment with OCR, OFA or UTX. Each point represents one patient, black bars show median and interquartile range. Numbers on the bottom refer to the number and percentage of individuals with reactive CD4^+^ or CD8^+^ T cells above the respective detection limits, which are indicated by the horizontal dotted line (0.03% for EBV-specific CD4^+^ T cells and 0.06% for EBV-specific CD8^+^ T cells, respectively). T-cell frequencies were compared between the different groups using Kruskal-Wallis tests with Dunn´s post-test. EBNA1, EBV nuclear antigen; IFNγ, interferon gamma; MS, multiple sclerosis; OCR, ocrelizumab; OFA, ofatumumab; UTX, ublituximab; SEB, *Staphylococcus aureus* enterotoxin B; VCA, virus capsid antigen

A subgroup analysis of pwMS on BCDT was conducted to investigate the effect of the B-cell depleting agents OCR, OFA, and UTX on the EBV-specific immune parameters. EBV-specific CD8^+^ T-cell frequencies showed significant differences among the patients receiving the various drugs (*p* = 0.0002), whereas polyclonal CD8^+^ T-cell levels did not differ between the groups (*p* = 0.760). Post-hoc analysis indicated the most pronounced depleting effect in patients on OCR with significantly lower median frequencies of specific CD8^+^ T cells (0.08% (IQR 0.19%) compared to the OFA group (0.54% (IQR 1.14%); *p* < 0.0001, Fig. 3A). The effect persisted even when PPMS patients from the OCR group were excluded from the analysis (overall *p* = 0.0004; post hoc OCR vs. OFA *p* = 0.0003). Patients treated with UTX also showed lower frequencies than those in the OFA group without reaching statistical significance in the post-hoc testing. The percentages of EBV-specific or SEB-reactive CD4^+^ T cells revealed no significant differences regarding the various BCDT agents (Fig. 3B). Regarding antibody titers, EBNA1-IgG were not differentially affected by the three drugs (Fig. 3C), whereas VCA-IgG titers differed with significantly higher titers in patients receiving OFA as compared to patients on OCR (Fig. 3D).

A multivariable regression analysis for EBV-specific cellular and humoral immune parameters adjusted for B-cell depleting agent, treatment duration, age and sex confirmed significantly higher levels of EBV-specific CD8^+^ T cells and VCA-IgG in patients in the OFA group (Table 3). Treatment duration showed a trend towards lower EBV-specific CD8^+^ T-cell frequencies, but did not reach statistical significance (*p* = 0.071, Table 3).

**Table 3:** Multivariable regression analyses for EBV-specific cellular and humoral immune parameters in pwMS on BCDT related to B-cell depleting agents and duration of therapy

Dependent variables^a^		EBV-specific CD8^+^ T cells		EBV-specific CD4^+^ T cells		anti-EBNA1 IgG		anti-VCA IgG	
Confounders		Estimate (95% CI)	*p*-value	Estimate (95% CI)	*p*-value	Estimate (95% CI)	*p*-value	Estimate (95% CI)	*p*-value
B-cell depleting agent	OCR [ref]	1		1		1		1	
	OFA	0.679 (0.256–1.102)	**0.002**	-0.017 (-0.237–0.203)	0.876	-0.067 (-0.376–0.242)	0.663	0.247 (0.010–0.483)	**0.042**
	UTX	0.129 (-0.420–0.678)	0.638	-0.048 (-0.334–0.237)	0.735	0.080 (-0.321–0.480)	0.691	0.112 (-0.196–0.419)	0.468
Duration of treatment		-0.0003 (-0.0006–0.00002)	0.071	0.00004 (-0.0001–0.0002)	0.602	-0.000002 (-0.0002–0.0002)	0.981	-0.00004 (-0.0002–0.0001)	0.620
Age		0.003 (-0.0009–0.016)	0.606	0.0001 (-0.007–0.006)	0.967	0.0003 (-0.009–0.009)	0.946	0.003 (-0.004–0.010)	0.380
Sex	male [ref]	1		1		1		1	
	female	-0.164 (-0.513–0.186)	0.352	-0.076 (-0.253–0.101)	0.393	0.074 (-0.175–0.322)	0.555	0.145 (-0.046–0.336)	0.133

Shown are *p*-values of multivariable linear regression analyses with log(10) transformed values; ^a^parameters refer to EBV-specific CD4 and CD8 T cells, and EBNA1 and VCA-IgG, with controls, and male persons as a reference for categorical parameters; OCR, ocrelizumab, OFA, ofatumumab, UTX, ublituximab; 95% CI, 95% confidence interval

Bold text indicates significant *p*-values (<0.05)

### Similar levels of EBV-specific T cells and antibodies in treatment-naïve pwMS as in controls

**Fig. 4:**
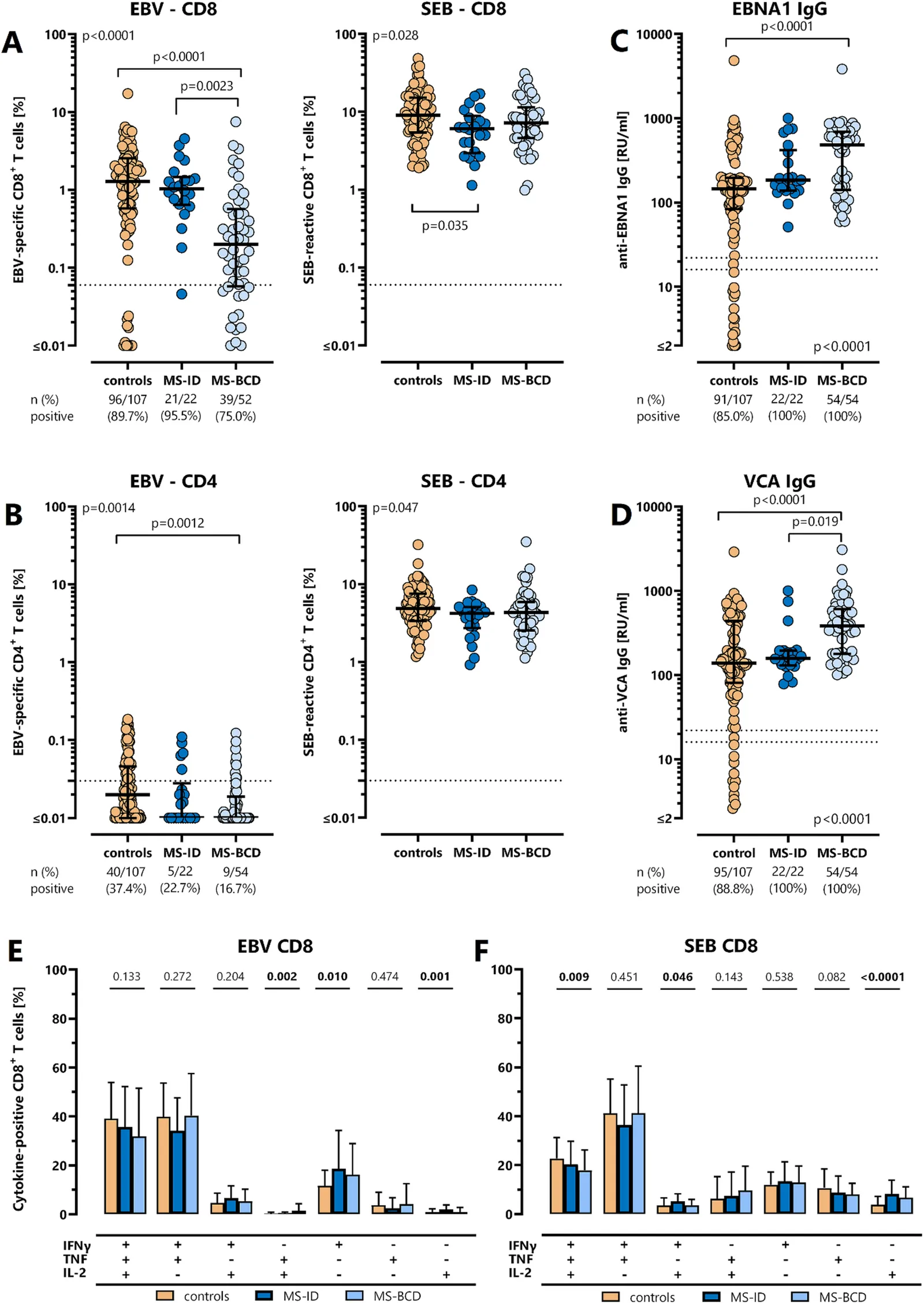
Similar levels of EBV-specific T cells and antibodies in treatment-naïve pwMS as in controls and preserved T-cell functionality across pwMS and controls. The levels of EBV-specific and SEB-reactive (**A**) CD8^+^ and (**B**) CD4^+^ T cells, as well as (**C**) EBNA1- and (**D**) VCA-specific IgG-levels are stratified for controls, treatment-naïve people with MS (MS-ID), and pwMS undergoing treatment with BCDT (MS-BCD). Each point represents one patient, black bars show median and interquartile range. Numbers on the bottom refer to the number and percentage of individuals with reactive CD4^+^ or CD8^+^ T cells above the respective detection limits, which are indicated by the horizontal dotted line (0.03% for EBV-specific CD4^+^ T cells and 0.06% for EBV-specific CD8^+^ T cells, respectively). T-cell frequencies were compared between the different groups using Kruskal-Wallis tests with Dunn´s post-test. (**E**) EBV-specific or (**F**) SEB-reactive CD8^+^ T cells from the three groups were analyzed for expression of IFNγ, TNF, and IL-2 alone or in combination. All samples from all individuals were analyzed, but only samples with at least 30 cytokine-expressing CD8^+^ T cells after subtraction of reactive cells of the negative control stimulation were considered to ensure robust statistical analyses of cells distributed to the seven subpopulations (*n* = 72 controls, *n* = 20 MS-ID, and *n* = 28 MS-BCD for EBV-specific CD8^+^ T cells, and *n* = 76 controls, *n* = 22 MS-ID, and *n* = 52 MS-BCD for SEB-reactive CD8^+^ T cells). Bars represent means and standard deviations. Comparisons were performed using a one-way ANOVA test. BCDT, B-cell depleting therapy; IFNγ, interferon gamma; pwMS, people with multiple sclerosis; IL-2, interleukin 2; SEB, *Staphylococcus aureus* enterotoxin B; TNF, tumor necrosis factor

To assess whether the observed effects were associated with BCDT or with MS, an additional cohort of 22 people with MS at the time of initial diagnosis were enrolled, who had not yet started immunomodulatory therapy at the time of inclusion (MS-ID; 20 female, 2 male; mean age 33.4±11.1 years, median EDSS 2.0 (IQR 1.0), median CD19^+^ cells 11.5% (IQR 7.3%), median serum IgG 1027 (IQR 367) mg/dl). As shown in Fig. 4A and B, EBV-specific CD8^+^ and CD4^+^ T-cell levels after stimulation with the peptide mix in MS-ID did not differ from healthy controls. However, among pwMS, MS-ID demonstrated significantly higher percentages of EBV-specific CD8^+^ T cells compared with the MS-BCD group. These effects were EBV-specific, as differences in SEB-reactive T cells between the three groups were less pronounced, irrespective of whether CD8^+^ or CD4^+^ T cells were taken into consideration. As with EBV-specific T cells, levels of specific antibodies in MS-ID were similar in magnitude as in controls, and numerically lower than in the MS-BCD group. While the difference in anti-EBNA1-IgG levels between MS-ID and MS-BCD groups did not reach statistical significance in the post-hoc analysis (Fig. 4C), anti-VCA-IgG titers in untreated pwMS were significantly lower (*p* = 0.019, Fig. 4D). Similar results were obtained when limiting the analyses to seropositive controls (Supplementary Figs. 3 and 5).

Further evaluation of the functional characteristics of T cells determined by cytokine expression profiling revealed that the majority of EBV-specific CD8^+^ T cells from controls and both patient groups exhibited preserved polyfunctional properties characterized by the simultaneous expression of IFNγ, IL-2, and TNF, or were double positive, displaying simultaneous expression of IFNγ and TNF. Despite some differences in the less abundant subpopulations, the cytokine expression profile was largely similar in the three groups (Fig. 4E). The profile of SEB-reactive CD8 T cells was distinct from EBV-specific cells with highest levels of IFNγ/TNF-dual positive followed by triple positive cells, and showed some subtle differences between the groups with less polyfunctional triple positive CD8^+^ T cells in the MS-BCD group (Fig. 4F).

### Different correlation matrices of EBV-specific antibodies and T cells in controls and pwMS

**Fig. 5:**
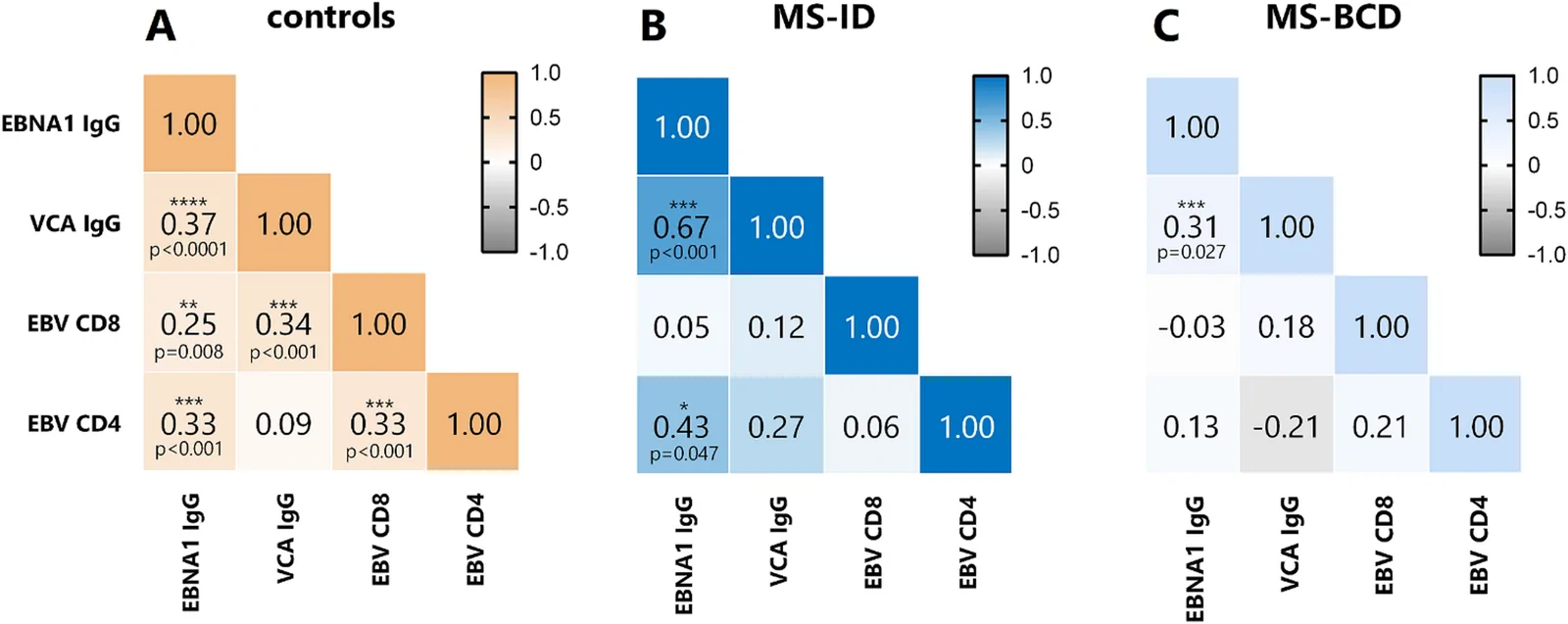
Different correlation matrices of EBV-specific antibodies and T cells in controls and pwMS. Correlation matrices displaying associations between EBV-specific CD8^+^ and CD4^+^ T-cell levels, as well as EBNA1- and VCA-IgG levels in (**A**) controls, (**B**) pwMS at initial diagnosis (MS-ID), and (**C**) pwMS receiving BCDT (MS-BCD). Correlations were calculated from log(10) transformed values according to Spearman with correlation coefficients visualized by a color code. BCDT, B-cell depleting therapy; EBNA1, EBV nuclear antigen; IFNγ, interferon gamma; pwMS, people with multiple sclerosis; VCA, virus capsid antigen

We finally analyzed whether EBV-specific cellular and humoral immune parameters correlated in the three groups. As shown in the correlation matrices in Fig. 5A, all tested parameters except EBV-specific CD4^+^ T cells and VCA-IgG showed a significant correlation in controls. In contrast, both untreated and treated pwMS showed a correlation between EBNA1- and VCA-IgG, whereas EBV-specific CD4^+^ T cells and EBNA1-IgG were found to correlate in untreated pwMS only (Fig. 5B and C). Detailed information on the correlation parameters including p-values and 95% confidence intervals are summarized in Supplementary Table 4.

A multivariable regression analysis for EBV-specific cellular and humoral immune parameters among the three groups adjusted for age and sex showed that being a pwMS on B-cell depleting therapy remained the only factor influencing EBV-specific CD4^+^ and CD8^+^ T-cell levels (Table 4). Likewise, this also had a significant effect on EBNA1- and VCA-IgG titers, although being an untreated pwMS or age were additional confounders affecting EBNA1-IgG levels, and VCA-IgG levels were also confounded by age and sex (Table 4).

**Table 4:** Multivariable regression analyses for EBV-specific cellular and humoral immune parameters

Dependent variables^a^		EBV-specific CD8^+^ T cells		EBV-specific CD4^+^ T cells		anti-EBNA1 IgG		anti-VCA IgG	
**Confounders**		Estimate (95% CI)	*p*-value	Estimate (95% CI)	*p*-value	Estimate (95% CI)	*p*-value	Estimate (95% CI)	*p*-value
Group	control [ref]	1		1		1		1	
	MS-ID	0.117 (-0.218–0.452)	0.491	-0.098 (-0.269–0.074)	0.263	0.394 (0.120–0.669)	**0.005**	0.207 (-0.027–0.441)	0.083
	MS-BCD	-0.664 (-0.895 – -0.433)	**< 0.0001**	-0.205 (-0.322 – -0.088)	**0.0007**	0.487 (0.300–0.674)	**< 0.0001**	0.428 (0.269–0.587)	**< 0.0001**
Age		0.006 (-0.0007–0.012)	0.082	0.001 (-0.003–0.004)	0.680	0.007 (0.002–0.012)	**0.006**	0.011 (0.006–0.015)	**< 0.0001**
Sex	male [ref]	1		1		1		1	
	female	-0.074 (-0.289–0.141)	0.496	-0.069 (-0.179–0.040)	0.214	0.070 (-0.106–0.245)	0.434	0.218 (0.068–0.367)	**0.005**

Shown are *p*-values of multivariable linear regression analyses with log(10) transformed values; ^a^parameters refer to EBV-specific CD4 and CD8 T cells, and EBNA1 and VCA-IgG, with controls, and male persons as a reference for categorical parameters; MS-BCD, people with MS on B-cell depleting therapy, MS-ID, people with MS at initial diagnosis; 95% CI, 95% confidence interval

Bold text indicates significant *p*-values (<0.05)

### Study population of healthy controls and pwMS on B-cell depleting therapy

## Discussion

In the present study, significantly lower levels of EBV-specific CD8^+^ T cells in pwMS undergoing BCDT were demonstrated. These levels were significantly lower than those observed in both a healthy control group and a group of untreated patients with primary diagnosis of MS, indicating that the observed effects should be regarded as treatment-associated rather than disease-associated. Similar differences were also observed for EBV-specific CD4^+^ T cells, but these results should be considered as exploratory as the percentage of reactive cells as detected with the peptide mix was below the limit of detection in most individuals. Unlike in controls, humoral EBV-specific immunity such as anti-EBNA1-IgG and anti-VCA-IgG levels were detectable in 100% of pwMS even under BCDT. Antibody levels in patients on BCDT were even significantly higher than those in healthy controls and treatment-naïve pwMS, highlighting the EBV-associated pathology in pwMS.

It has been suggested that EBV-specific CD8^+^ T cells play a pivotal role in the pathophysiology of MS. They are enriched in the cerebrospinal fluid and in brain lesions of pwMS, and are more prevalent than other virus- or autoantigen-specific CD8^+^ T cells [29–32]. It is discussed that persistent EBV-infection of B cells that can penetrate the CNS leads to an increased, dysregulated autoimmune response involving cytotoxic CD8^+^ T cells, resulting in continuous inflammation and ‘bystander’ damage to brain tissue [33]. Furthermore, numerous studies have explored the pathophysiological role of EBV-specific CD4^+^ T cells reactive to latent antigens such as EBNA1 [34], and more recent data further suggest an additional influence of CD4^+^ T cells reactive against lytic antigens [16]. As CD20^+^ B cells are the main reservoir of EBV, their depletion represents a therapeutic target to reduce autoinflammatory processes in line with the above hypothesis [24, 35]. Previous longitudinal studies have shown that BCDT leads to a reduction in EBV-specific T cells in pwMS in the blood, as well as to a decrease in the proliferation of these cells [22, 36]. EBV-specific T cells appear to decline in later stages of the disease and in cases with a progressive course [15, 37]. This is consistent with the finding that PPMS patients in our cohort tended to have lower T-cell frequencies than pwRMS. However, previously published cohorts were small and no comparison was made with healthy controls. We demonstrated that the levels of specific T cells in pwMS treated with BCDT was significantly lower than the levels seen in healthy controls, and showed an association of lower levels with higher EDSS and increasing time of BCDT. This decrease may result from a reduced antigen load following the depletion of infected CD20^+^ B cells, thereby leading to an indirect reduction in antigen-specific T-cell responses. It indicates the exceptional efficacy of the therapy with regard to EBV-specific T-cell immunity. The effect does not appear to be a general effect on T-cellular immunity, as overall T-cell reactivity as measured following polyclonal activation remains unaffected by BCDT. This finding is also consistent with the results of recently published vaccination studies, showing that the induction of SARS-CoV-2 spike-specific T-cell responses was also unaffected by BCDT or even stronger for CD8 T cells when compared to healthy controls [38]. Likewise, despite reduced levels of EBV-specific T cells during BCDT, both EBV-specific and polyclonal T-cell functionality appears to be largely preserved with the remaining T cells being predominantly polyfunctional in both patients and controls.

Previous work on the role of BCDT on EBV-specific immunity has focused solely on people with MS receiving OCR [22, 36]. We now show that the different BCDT agents had varying effects on the suppression of EBV-specific T cells, with significantly stronger suppression observed with OCR (or UTX) than with OFA. One explanation could be that OFA primarily acts via complement-dependent cytotoxicity, while OCR and UTX mainly act via antibody-dependent cell-mediated cytotoxicity [39]. However, given the limited sample sizes in the subgroups, this is only an explorational finding; these differences warrant further attention and must be confirmed in future studies.

So far, only a few studies have been conducted regarding the influence of BCDT on EBV-specific antibodies [20, 21, 23, 40]. However, the sample size in these was low, and the studies primarily focused on anti-EBNA1-IgG, while concomitant knowledge on antibodies towards other EBV-derived antigens such as anti-VCA-IgG is limited. Overall, it appears that EBV-specific antibody levels are preserved or rather decrease slightly during therapy; however, patients in the various studies differed in treatment modalities, and had in part no control group. Detectable antibodies are overall consistent with other studies which have shown that pre-existing antibody immunity often persists in patients receiving anti-CD20-directed BCDT. This phenomenon may be attributable to the presence of long-lived bone marrow plasma cells that are independent of memory B cells targeted by B-cell depleting agents [41–43]. However, our findings even show that patients on BCDT had significantly higher EBV-specific antibody levels than controls or patients with newly diagnosed MS. As EBV-load also appears to decrease under BCDT [16], the relative increase in antibody levels may be explained by reduced antibody consumption due to lower levels of viral replication. Other explanations consist in the higher age and disease progression in the BCDT cohort. Given these persistently increased levels of EBV-specific antibodies in pwMS undergoing BCDT in the face of a very high efficacy of the therapy in terms of disease activity, it can be speculated that antibody-mediated EBV immunity may play a less pronounced role in the course of the disease than the EBV-specific cellular immunity.

### Strengths and limitations

To our knowledge, this is the largest cohort to date in which the effects of three different anti-CD20-depleting agents on EBV-specific immune parameters have been analyzed. Moreover, data directly comparing EBV-specific CD8^+^ T cells and antibody levels in pwMS with and without BCDT with those of healthy controls were so far limited. The same applies to the functional characterisation of these specific T cells. Longitudinal data from disease onset throughout therapy including analyses of specific cells from blood and CSF will be of interest to further characterize the role and spatial distribution of EBV-specific immunity in the pathogenesis and course of MS. While our results on EBV-specific CD8 + T cells can be considered robust due to a predominant represenation of MHC class I binding peptides, our data on EBV-specific CD4^+^ T cells should be interpreted with caution, as the CD4 T-cell arm of the assay rests on a very narrow set of peptides. In addition, given that most persons had a response below the lower limit of detection, participants may lack alleles required to present this more limited set of peptides. CD4 T-cell results should therefore considered as exploratory, and future studies should use stimuli that allow for a more comprehensive detection of EBV-specific CD4^+^ T cells. Moreover, future studies could also include comprehensive analyses of immune responses to other persistent pathogens, such as cytomegalovirus or varicella zoster virus to further disentangle the specificity of the effects regarding other herpesviruses. Although we have included three different B-cell depleting agents, the study is limited by the absence of a group of pwMS with other treatments such as natalizumab or oral drugs, which restricts the attribution of the observed differences specifically to anti-CD20 treatment. In addition, the comparisons between OCR, OFA, and UTX should be interpreted cautiously because of the small subgroup sizes and potential confounding by phenotype. Finally, it should be noted that the most significant genetic risk factor (the HLA-DR15 haplotype, which synergistically increases the risk of MS in conjunction with EBV [44]) was not assessed and HLA status in general was not known.

## Conclusions

BCDT is associated with selectively lower EBV-specific T-cell levels, while general T-cell functionality was preserved. In contrast, the humoral response to EBV is far less affected, with significantly elevated levels of anti-EBNA1 and anti-VCA antibodies. The virus plays a role in the pathophysiology of MS, and BCDT has been found to be highly efficacious. Considering our findings, the significance of EBV-specific T-cell reactivity may be greater than that of humoral immunity. Given the differential effects of B-cell depleting agents on reducing EBV-specific T-cell levels, future studies should elucidate whether the depletion of specific T cells is associated with the clinical effectiveness of the drugs. Thus, EBV-specific immunomonitoring may be developed to facilitate personalized treatment and assignment of drug regimens.

## Supplementary Information

Supplementary Material 1.

## Data Availability

No datasets were generated or analysed during the current study.

## Abbreviations

BCDT: B-Cell depleting therapy
CNS: Central nervous system
EBNA1: EBV nuclear antigen 1
EBV: Epstein-Barr virus
EDSS: Expanded disability status scale
ELISA: Enzyme-linked immunosorbent assay
IFNγ: Interferon gamma
IgG: Immunoglobulin G
IL-2: Interleukin-2
IQR: Interquartile range
ml: Milliliter
MS: Multiple sclerosis
OCR: Ocrelizumab
OFA: Ofatumumab
PPMS: Primary progressive multiple sclerosis
pwMS: People with multiple sclerosis
RMS: Relapsing multiple sclerosis
RU: Relative units
TNF: Tumor necrosis factor
UTX: Ublituximab
VCA: Virus capsid antigen
