# Supplementary material for: The influence of anti-CD20-directed B cell depletion across different agents on Epstein-Barr virus-specific humoral and cellular immunity in patients with multiple sclerosis
Source: J Neuroinflammation. 2026 Sep 28;23:326. doi: 10.1186/s12974-026-04062-0 (PMC13621653; doi:10.1186/s12974-026-04062-0)
Supplement: Supplementary file 1 [file 12974_2026_4062_MOESM1_ESM.docx]

# Supplementary Data

# The influence of anti-CD20-directed B cell depletion across different agents on Epstein-Barr virus-specific humoral and cellular immunity in patients with multiple sclerosis

This supplement includes 5 supplementary figure and 4 supplementary tables.

# Supplementary figures

## Supplementary figure 1

**Supplementary figure 1: No difference in results obtained by two different flow-cytometers.** The control group was analysed on two different flow-cytometers (n=61 (mean age 39.9±16.0 years) on FACS Canto II, and n=46 (mean age 56.8±16.7 years) on FACSymphony A3). All patients with multiple sclerosis were also analysed on FACSymphony A3 (see figure 2). The percentage of EBV-specific or SEB-reactive **(A)** CD8^+^ T cells or **(B)** CD4^+^ T cells did not differ between samples analysed on the two flow-cytometers. EBV, Epstein-Barr virus; SEB, *Staphylococcus aureus* enterotoxin B.

## Supplementary figure 2

**Supplementary Figure 2: Gating strategy for identification of antigen-specific CD8+ and CD4^+^ T cells after stimulation**. **(A)** After stimulation of whole blood, lymphocytes were identified by CD45+ leukocytes and backgating of CD3^+^ cells combined with signals for size (FSC) and granularity (SSC). **(B)** To exclude doublets and cell aggregates, height and area signals of FSC and the BV421 channel (not assigned to an antibody) were used. As exemplified for stimulation with DMSO **(C-I),** EBVpan **(C-II)** or SEB **(C-III)**, antigen-specific CD8^+^ T cells, among single CD3^+^ lymphocytes, were identified by co-expression of the activation marker CD69 and the cytokine IFNγ. The same gating strategy was applied to identify antigen-specific CD4^+^ T cells. Boolean gating for cytokine profiling based on staining of IFNγ, IL-2 and TNF is shown that was used to identify subpopulations of EBV- or SEB-reactive CD8^+^ T cells expressing all three cytokines (triple+), two cytokines or one cytokine only. Cytokine profiles were calculated as percentage of subpopulation from all cytokine expressing cells. EBV-specific profiles were calculated after correction for the negative control (DMSO). Boxes were used as gates to quantify the percentage of CD69^+^/IFNγ^+^ CD8^+^ or CD4^+^ T cells.

## Supplementary figure 3

**Supplementary figure 3: Higher levels of EBV-specific IgG as compared to RSV- and CMV-IgG in pwMS on BCDT.** Levels of **(A)** anti-EBNA1-IgG, **(B)** anti-VCA-IgG **(C)** anti-RSV-IgG and **(D)** anti-CMV-IgG were compared between controls, pwMS with initial diagnosis (MD-ID, see figure 4), and pwMS on BCDT (MS-BCD). Each point represents one participant. The black lines refer to median and interquartile ranges. Antibody levels are calculated in relative units per ml. Only seropositive individuals are shown. Numbers on the bottom refer to the number and percentage of individuals with antibody levels above the detection limit for positivity as indicated by the dotted lines referring to positive, intermediate and negative titers, with the denominator referring to all individuals in the respective groups. Groups were compared using the Kruskal Wallis test with Dunn´s post test. CMV, cytomegalovirus, BCDT, B-cell depleting therapy; EBNA1, EBV nuclear antigen 1; IgG, immunoglobulin G; MS-ID, pwMS at initial diagnosis; MS-BCD, pwMS on B-cell depleting therapy; pwMS, people with multiple sclerosis; RSV, respiratory syncytial virus; VCA, virus capsid antigen.

## Supplementary figure 4

**Supplementary figure 4: Negative control stimulation (DMSO) and EBV-specific stimulation in EBNA-1-/VCA-IgG seropositive and seronegative individuals. (A)** Paired display of reactive CD8^+^ and CD4^+^ T cells after stimulation with DMSO and EBVpan peptide mix (EBV), respectively, in nine EBNA1/VCA-IgG seronegative healthy controls, and percentage of EBV-specific CD8 and CD4 T cells defined as CD69^+^IFNγ^+^ T cells after subtraction of reactive cells after DMSO negative control stimulation (right panel). The lower limit of detection of 0.06% for CD8^+^ T cells and 0.03% for CD4^+^ T cells is indicated as stippled lines. Paired display of reactive **(B)** CD8^+^ and **(C)** CD4^+^ T cells after stimulation with DMSO and EBVpan peptide mix (EBV) in EBNA1/VCA-IgG seropositive controls and pwMS (MS-ID and MS-BCD). Statistical analyses were carried out using the non-parametric Wilcoxon matched pairs test. EBNA1, EBV nuclear antigen 1; IgG, immunoglobulin G; IFNγ, interferon gamma; LOD, lower limit of detection; pwMS, people with multiple sclerosis; MS-ID, pwMS at initial diagnosis; MS-BCD, pwMS on B-cell depleting therapy; SEB, *Staphylococcus aureus* enterotoxin B, VCA, virus capsid antigen.

-

## Supplementary figure 5

**Supplementary figure 5: Lower levels of EBV-specific CD8^+^ and CD4^+^ T cells in pwMS on BCDT compared to EBV-EBNA-1/VCA seropositive controls.** EBV-specific and SEB-reactive **(A)** CD8^+^ T cells and **(B)** CD4^+^ T cells were compared between EBV-EBNA1 and/or VCA IgG seropositive controls (n=98), pwMS with initial diagnosis (MS-ID, n=22, all seropositive), and pwMS on BCDT (MS-BCD, n=54, all seropositive). Each point represents T-cell frequencies of one participant. The black lines refer to median and interquartile ranges. Numbers on the bottom refer to the number and percentage of seropositive individuals with reactive CD4^+^ or CD8^+^ T cells above the respective detection limits, which are indicated by the horizontal dotted line (0.03% for EBV-specific CD4^+^ T cells and 0.06% for EBV-specific CD8^+^ T cells, respectively). Levels of CD8^+^ and CD4^+^ T cells toward EBVpan peptide mix and the polyclonal stimulus SEB were compared between the groups using the Kruskal Wallis test with Dunn´s post test. The samples of two MS-BCD patients were excluded from the EBV-specific CD8 T-cell analyses as indeterminate due to high non-specific background reactivity in the control stimulation. BCDT, B-cell depleting therapy; EBNA1, EBV nuclear antigen 1; IgG, immunoglobulin G; IFNγ, interferon gamma; MS-ID, pwMS at initial diagnosis; MS-BCD, pwMS on B-cell depleting therapy; pwMS, people with multiple sclerosis; SEB, *Staphylococcus aureus* enterotoxin B; VCA, virus capsid antigen.

# Supplementary Tables

## Supplementary table 1: Composition of the EBVpan peptide mix (PM-pan-EBVselect).

| **Protein** | **Gene** | **Number of peptides** | **MHC class I** | **MHC class II** |
| --- | --- | --- | --- | --- |
| Single-stranded DNA-binding protein | BALF2 | 1 | x |  |
| DNA polymerase catalytic subunit | BALF5 | 1 | x | x |
| Secreted protein BARF1 | BARF1 | 6 | x |  |
| Portal protein | BBRF1 | 1 | x |  |
| Viral interleukin-10 homolog | BCRF1 | 1 | x |  |
|  | BFRF3 | 1 | x |  |
| Apoptosis regulator BHRF 1 | BHRF1 | 1 | x | x |
| Glycoprotein BILF2 | BILF2 | 3 | x |  |
| Envelope glycoprotein GP350 | BLLF1 | 1 | x |  |
| mRNA export factor ICP27 homolog | BMLF1 | 2 | x |  |
| DNA polymerase processivity factor BMRF1 | BMRF1 | 3 | x |  |
| Major tegument protein | BNRF1 | 2 | x |  |
| Large tegument protein deneddylase | BPLF1 | 1 | x |  |
| Replication and transcription activator | BRLF1 | 6 | x |  |
| Uncharacterized protein | BTRF1 | 1 | x |  |
| Trans-activator protein | BZLF1 | 10 | x |  |
| Epstein-Barr nuclear antigen 1 | EBNA1 | 16 | x | x |
| Epstein-Barr nuclear antigen 2 | EBNA2 | 8 | x | x |
| Epstein-Barr nuclear antigen 3 | EBNA3 | 12 | x | x |
| Epstein-Barr nuclear antigen 4 | EBNA4 | 9 | x |  |
| Epstein-Barr nuclear antigen 6 | EBNA6 | 13 | x | x |
| Epstein-Barr nuclear antigen leader protein | EBNA-LP | 1 | x |  |
| Envelope glycoprotein B | gB | 2 | x |  |
| Envelope glycoprotein H | gH | 3 | x |  |
| Envelope glycoprotein L | gL | 1 | x |  |
| Latent membrane protein 1 | LMP1 | 10 | x |  |
| Latent membrane protein 2 | LMP2 | 19 | x | x |

Peptides are provided as mix and were purchased from jpt Berlin, Germany.

## Supplementary table 2: Human MHC class I and MHC class II allele coverage of the PM-pan-EBVselect peptide mix

| **HLA** | **Alleles** |
| --- | --- |
| HLA-A | HLA-A*02:01, HLA-A*02:02, HLA-A*02:03, HLA-A*02:04, HLA-A*02:06, HLA-A*02:07, HLA-A*02:09, HLA-A*03:01, HLA-A*11:01, HLA-A*23:01, HLA-A*24:02, HLA-A*24:03, HLA-A*29:01, HLA-A*30, HLA-A*68:01, HLA-A*68:02, HLA-A*69:01 |
| HLA-B | HLA-B*07:02, HLA-B*08:01, HLA-B*08:02, HLA-B*15:01, HLA-B*15:02, HLA-B*18:01, HLA-B*27:02, HLA-B*27:04, HLA-B*27:05, HLA-B*27:06, HLA-B*27:09, HLA-B*35:01, HLA-B*35:02, HLA-B*35:03, HLA-B*35:08, HLA-B*37:01, HLA-B*38:01, HLA-B*39:06, HLA-B*40:01, HLA-B*40:02, HLA-B*44:02, HLA-B*44:03, HLA-B*44:05, HLA-B*45:01, HLA-B*46:01, HLA-B*51, HLA-B*53:01, HLA-B*55:01, HLA-B*57:01, HLA-B*58:01, HLA-B*60, HLA-B*61, HLA-B*62 |
| HLA-C | HLA-C*03:03, HLA-C*03:04, HLA-C*08:02, HLA-C*02, HLA-C*06 |
| HLA-DR/-DP/-DQ | HLA-DP, HLA-DQ*02, HLA-DQ*05, HLA-DQB1*06:01, HLA-DR, HLA-DRB1*01:01, HLA-DRB1*01:03, HLA-DRB1*03:01, HLA-DRB1*04:01, HLA-DRB1*04:03, HLA-DRB1*04:04, HLA-DRB1*04:06, HLA-DRB1*07:01, HLA-DRB1*09:01, HLA-DRB1*11:01, HLA-DRB1*15:01, HLA-DRB1*15:02, HLA-DRB1*16:02, HLA-DRB1*17, HLA-DRB3, HLA-DRB5*01:01 |

Information provided by jpt Berlin, Germany.

## Supplementary table 3: Antibodies used for flow cytometric analysis.

| **Antigen** | **Conjugate** | **Clone** | **Isotype** | **Working concentration^a^** | **Catalogue number** | **RRID^b^** |
| --- | --- | --- | --- | --- | --- | --- |
| CD3 | FITC | SK7 | IgG1 κ | 1:100^d^ | 345763 | AB_2811220 |
| CD4 | APC-H7 | SK3 | IgG κ | 1:33.3 | 641398 | AB_1645732 |
| CD4 | APC-H7 | SK3 | IgG κ | 1:66.7^d^ | 641398 | AB_1645732 |
| CD45 | BUV805 | HI30 | IgG1 κ | 1:200^d^ | 612891 | AB_2870179 |
| CD8 | PerCP | SK1 | IgG1 | 1:12.5 | 345774 | AB_2868802 |
| CD8 | BUV395 | RPA-T8 | IgG1 κ | 1:200^d^ | 563795 | AB_2722501 |
| CD28^c^ |  | L293 | IgG1 κ | 1µg/ml | 348040 | AB_400367 |
| CD49d^c^ |  | 9F10 | IgG1 κ | 1µg/ml | 555501 | AB_396068 |
| CD69 | PE-Cy7 | L78 | IgG1 κ | 1:50 | 335792 | AB_1937286 |
| CD69 | RY775 | FN50 | IgG1 κ | 1:400^d^ | 571396 | AB_3686476 |
| IFNγ | FITC | 4S.B3 | IgG1 κ | 1:100 | 554551 | AB_395473 |
| IFNγ | BV480 | B27 | IgG1 κ | 1:66.7^d^ | 566100 | AB_2739503 |
| IL-2 | PE | MQ1-17H12 | IgG2a | 1:25.5 | 559334 | AB_397231 |
| IL-2 | RB705 | MQ1-17H12 | IgG2a κ | 1:200^d^ | 570624 | AB_3685902 |
| TNF | RY610 | MAb11 | IgG1 κ | 1:769^d^ | 571267 | AB_3686371 |
| TNF | V450 | MAb11 | IgG1 κ | 1:50 | 561311 | AB_10646031 |

^a^The concentration of the antibodies used for antigen-specific stimulation was indicated as a concentration in a final volume of 50µl (FACSCanto II) or 100µl (FACSymphony A3) staining solution; ^b^RRID, Research Resource ID; all antibodies are mouse anti-human except anti-IL-2, which are rat anti-human; ^c^non-conjugated costimulatory antibodies used during antigen-specific stimulation; ^d^values referring to FACSymphony A3; all antibodies were separately titrated in house to evaluate best concentrations for discrimination of negative and positive cell populations; panels including defined concentration of antibodies were tested together to confirm that identified antibody concentrations are also sufficient when compensations are required; all antibodies from BD Biosciences, Heidelberg, Germany.

## Supplementary table 4: Correlation parameters according to Spearman

|  |  | **Controls** | **MS-ID** | **MS-BCD** |
| --- | --- | --- | --- | --- |
| **Correlation of** |  | n=107* | n=22* | n=54* |
| EBNA1 IgG with  VCA IgG | r 95% CI p-value | **0.37 (0.19 - 0.53) <0.0001** | **0.67 (0.34 - 0.86) <0.001** | **0.31 (0.03 - 0.54) 0.027** |
| EBNA1 IgG with  EBV-specific CD8^+^ T cells | r 95% CI p-value | **0.25 (0.062 - 0.43) 0.008** | 0.05 (-0.39 - 0.47) 0.836 | -0.03 (-0.31 - 0.25) 0.835 |
| EBNA1 IgG with EBV-specific CD4^+^ T cells | r 95% CI p-value | **0.33 (0.15 - 0.50) <0.001** | **0.43 (-0.005 - 0.73) 0.047** | 0.13 (-0.15 - 0.40) 0.340 |
| VCA IgG with  EBV-specific CD8^+^ T cells | r 95% CI p-value | **0.34 (0.15 - 0.50) <0.001** | 0.12 (-0.33 - 0.53) 0.580 | 0.18 (-0.10 - 0.44) 0.190 |
| VCA with EBV-specific CD4^+^ T cells | r 95% CI p-value | 0.09 (-0.11 - 0.28) 0.368 | 0.27 (-0.18 - 0.63) 0.222 | -0.21 (-0.46 - 0.079) 0.143 |
| EBV-specific CD8^+^ T cells with EBV-specific CD4^+^ T cells | r 95% CI p-value | **0.33 (0.14 - 0.49) <0.001** | 0.06 (-0.38 - 0.48) 0.775 | 0.21 (-0.076 - 0.46) 0.137 |

*Correlation parameters for controls, MS-ID and MS-BCD as graphically displayed in figure 5; EBNA1, EBV nuclear antigen 1; IgG, Immunoglobulin G; pwMS, people with multiple sclerosis; MS-ID, pwMS with initial diagnosis; MS-BCD, pwMS on B-cell depleting therapy; VCA, virus capsid antigen.
